# Performance of Various Pelvic-Derived Samples for the Diagnosis of Female Genital Tract Tuberculosis by Conventional and Molecular Methods: A Systematic Review and Meta-Analysis

**DOI:** 10.7759/cureus.84016

**Published:** 2025-05-13

**Authors:** Seetu Palo, Sarita Rawat, Debadutta Mishra, Monica Mishra

**Affiliations:** 1 Department of Pathology and Laboratory Medicine, All India Institute of Medical Sciences, Bibinagar, Hyderabad, IND; 2 Department of Microbiology, Teerthanker Mahaveer University (TMU) Medical College, Moradabad, IND; 3 Department of Microbiology, KMC Medical College, Maharajganj, IND

**Keywords:** diagnostic accuracy, endometrial biopsy, female genital tuberculosis, genexpert mtb/rif, menstrual blood, polymerase chain reaction

## Abstract

Female genital tuberculosis (FGTB) is a challenging extrapulmonary manifestation of tuberculosis, often presenting with nonspecific symptoms and a paucibacillary profile, complicating diagnosis. This systematic review and meta-analysis evaluated the diagnostic performance of various pelvic-derived samples using conventional and molecular tests. A comprehensive literature search was conducted across multiple databases from inception up to August 2024, following the Preferred Reporting Items for Systematic Reviews and Meta-Analyses (PRISMA) guidelines. Studies assessing the sensitivity, specificity, or positivity rates of tests such as Ziehl-Neelsen (ZN) staining, culture, histopathology, polymerase chain reaction (PCR), and GeneXpert MTB/RIF on samples including endometrial biopsy, aspirates, menstrual blood, and peritoneal fluid were included. Meta-analysis using bivariate random-effects models was undertaken where feasible. Endometrial samples were the most commonly evaluated among the included studies. ZN staining and culture demonstrated high specificity (pooled specificity: 1.00) but poor sensitivity (ZN: 10%; culture: 23%). Histopathology exhibited variable sensitivity (2.56-75%) and high specificity (98%). PCR showed pooled sensitivity and specificity of 54% and 97%, respectively, with considerable heterogeneity. GeneXpert demonstrated excellent specificity (pooled 100%) but low sensitivity (14%). Menstrual blood and pelvic washings were explored with variable results; other sample types had limited diagnostic value. In conclusion, endometrial biopsy/aspirate remains the most suitable specimen for FGTB diagnosis. Molecular methods, particularly PCR, offer superior sensitivity over conventional tests, while GeneXpert's high specificity supports its role in exclusion. A multimodal diagnostic approach is recommended to enhance diagnostic yield, especially in resource-limited, high-TB-burden settings.

## Introduction and background

Tuberculosis (TB) remains a significant global health challenge, with approximately 6.4 million new TB cases documented worldwide. While pulmonary TB is the most common presentation, extrapulmonary TB accounts for a substantial proportion of cases. Female genital tuberculosis (FGTB) is an important form of extrapulmonary TB, accounting for 3-16% of extrapulmonary TB cases in endemic countries like India [1]. FGTB primarily affects women of reproductive age and presents with vague symptoms such as infertility, menstrual disorders, and chronic pelvic pain, making clinical diagnosis challenging [2]. Additionally, the paucibacillary nature of FGTB and the difficulty in obtaining adequate samples from deep-seated organs further complicate laboratory diagnosis [2]. The fallopian tubes are the most commonly affected site (95-100%), followed by the endometrium (50-80%), ovaries (20-30%), cervix (5-15%), and vagina/vulva (1%) [3]. Due to the varied manifestations and sites of involvement, different sampling methods and diagnostic techniques have been employed, with varying diagnostic yields. Early diagnosis and prompt treatment are essential to prevent irreversible reproductive damage.

Conventional methods, such as microscopy for acid-fast bacilli (AFB) and culture, have limited sensitivity in FGTB due to its paucibacillary nature [4]. Histopathological examination (HPE) showing granulomatous inflammation may support the diagnosis but lacks specificity. Molecular methods like polymerase chain reaction (PCR) and GeneXpert MTB/RIF assay have emerged as promising tools, but their performance varies depending on the sample type and processing methods [1]. This systematic review aims to evaluate and compare the diagnostic performance of various pelvic-derived samples (peritoneal fluid, endometrial biopsy, endometrial aspirate, menstrual blood, etc.) using different diagnostic methods (microscopy, culture, histopathology, PCR, GeneXpert MTB/RIF, etc.) for detecting FGTB. The findings will guide clinicians in selecting the most appropriate sample type and diagnostic method for FGTB diagnosis.

## Review

Methodology

This systematic review was conducted following the Preferred Reporting Items for Systematic Reviews and Meta-Analyses (PRISMA) guidelines, and the study protocol was registered in the International Prospective Register of Systematic Reviews (PROSPERO) with the registration number CRD42024508025. A comprehensive electronic literature search was performed using databases including PubMed, Scopus, ProQuest, and Google Scholar to identify relevant studies from inception to August 2024. The search strategy incorporated a combination of Medical Subject Headings (MeSH) and free-text terms related to "female genital tuberculosis", "diagnosis", and "diagnostic accuracy" (Appendix 1). Studies were selected based on predefined inclusion and exclusion criteria. Original research, short communications, and pre-print report articles evaluating the sensitivity and specificity or positivity rate of one or more diagnostic tests for FGTB were included. Exclusion criteria encompassed articles focusing on genitourinary TB, inclusive of male patients, case reports, case series, conference abstracts, review articles, letters to the editor, animal studies, and non-English publications.

Data extraction was performed independently by two authors (SR and MM) using a standardized data collection form. Extracted information included study characteristics (year of publication, geographic location, sample size), patient demographics (age, clinical presentation, mean duration of presentation), sample type (endometrial biopsy, endometrial aspirate, menstrual blood, pelvic washings, ovarian biopsy, etc.), diagnostic test employed (Ziehl-Neelsen (ZN) staining, culture, histopathology, PCR, GeneXpert MTB/RIF), reference standard used, and test performance metrics (true positives, false positives, true negatives, false negatives, sensitivity, specificity, positive predictive value, negative predictive value). The extracted data was cross-checked by two authors (SP and DM). The quality of included studies was assessed using the Quality Assessment of Diagnostic Accuracy Studies-2 (QUADAS-2) tool, and any discrepancies in study selection, data extraction, or quality assessment were resolved through discussion or consultation with a third reviewer.

Meta-analysis was conducted where sufficient data were available, using Meta-Disc® (Version 1.4, XI Cochrane Colloquium, Spain) [5]. The pooled sensitivity and specificity for each test type were calculated using a bivariate random-effects model. Both the Cochran Q statistic and I^2^ tests were employed to evaluate heterogeneity. Substantial heterogeneity among studies was inferred with a p-value below 0.05 or an I^2^ value of 50% or greater, while minimal heterogeneity was indicated by higher p-values or I^2^ values below 50%. The findings were synthesized narratively, where meta-analysis was not feasible due to data variability. Publication bias was assessed by Egger's regression test for funnel plot asymmetry and Rosenthal's fail-safe N using jamovi (Version 2.3.28, The jamovi project, Sydney, Australia) [6].

Results

**Figure 1:**
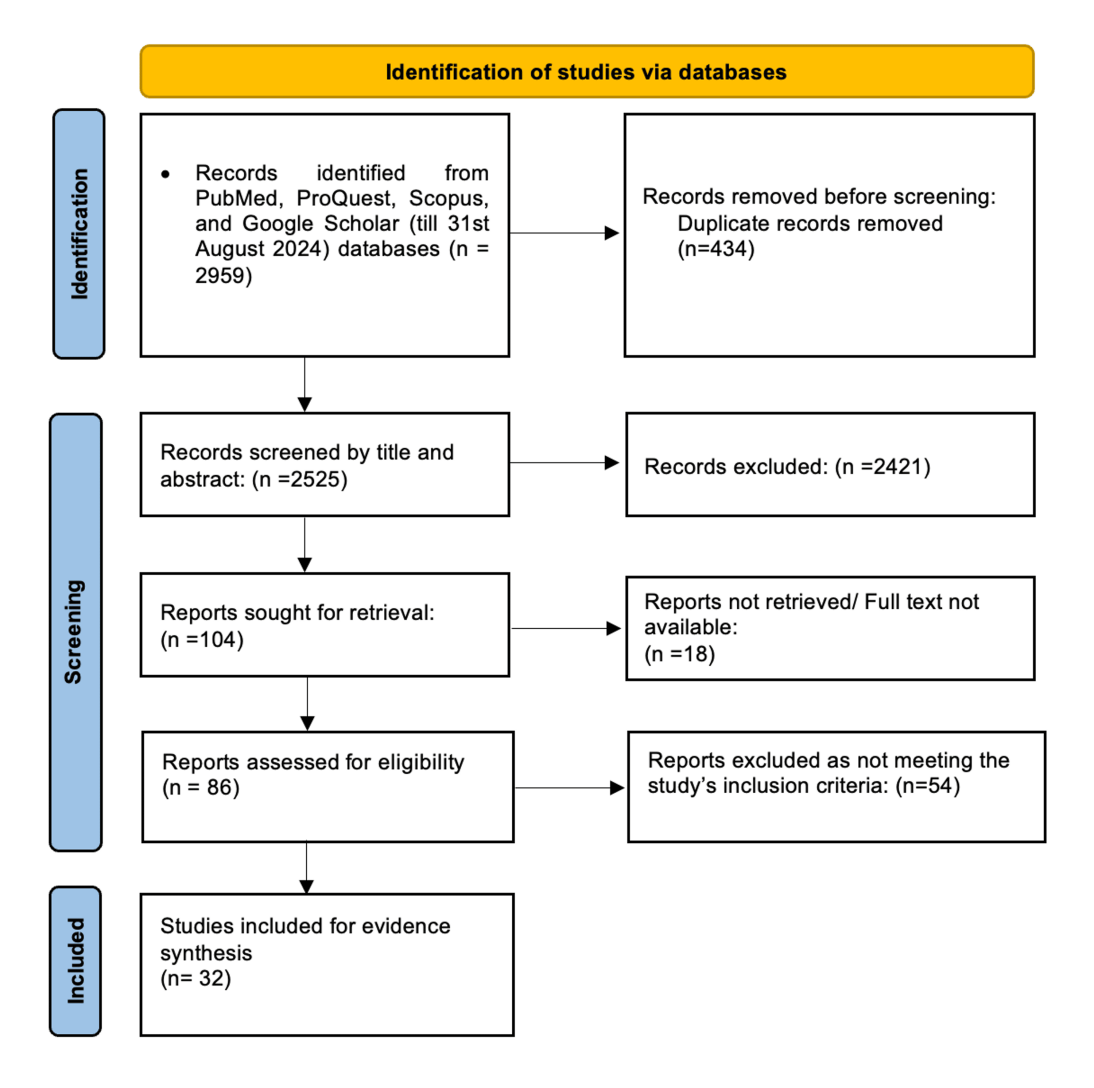
PRISMA flowchart of study selection PRISMA: Preferred Reporting Items for Systematic Reviews and Meta-Analyses

Figure 1 shows the PRISMA flow diagram depicting the study selection process (Appendix 2). A total of 2959 studies were identified through database searching. After removing duplicates, 2525 studies underwent title and abstract screening. After full-text screening, 32 studies met the inclusion criteria and were included in the qualitative synthesis. All the studies were from India with a high TB burden. All the studies included women presenting with infertility, with or without additional symptomatology such as pelvic pain, menstrual disorders, or suspected pelvic TB based on radiological findings.

Table 1 and Table 2 represent the positivity rate, sensitivity, and specificity of different diagnostic methods across studies evaluating TB detection in endometrial biopsy samples [7-33]. The diagnostic modalities included conventional tests such as AFB microscopy using ZN staining, culture for *Mycobacterium tuberculosis*, HPE for granulomatous inflammation, and molecular tests such as TB-PCR and GeneXpert. The positivity rates varied significantly across studies, influenced by factors such as sample size, diagnostic technique, and disease prevalence. Large-scale studies like Malhotra et al. with 555 participants and Sankar et al. with 620 participants reported notably high TB-PCR positivity as compared to that of conventional methods, reinforcing PCR's diagnostic advantage [16,21]. However, some studies, such as Pathak et al. and Kanade et al., showed inconsistencies, with culture and HPE detecting more cases than GeneXpert, likely due to variations in sampling techniques and test execution [12,19]. Overall, GeneXpert and TB-PCR generally showed higher positivity compared to conventional methods like AFB microscopy and HPE, highlighting their sensitivity.

**Table 1:** Positivity rates of conventional and molecular methods for detecting female genital tuberculosis in endometrial biopsy AFB: acid-fast bacilli; ZN: Ziehl-Neelsen; HPE: histopathological examination; TB-PCR: tuberculosis-polymerase chain reaction

Author	No. of participants	Age range in years	Number of samples tested positive				
			AFB microscopy/ZN staining	Culture	HPE	TB-PCR	GeneXpert
Ashwini et al. [7]	1754	20-45	-	-	-	24 (1745)	5 (1475)
Chaubey et al. [8]	194	19-38	-	-	-	112	-
Chaudhary et al. [9]	100	20-40	-	2	1	-	3
Dogra et al. [10]	100	-	-	4	7	15	-
Gajbhiye et al. [11]	50	18-40	2	1	-	4	-
Kanade et al. [12]	75	20-46	3	21	17	-	14
Kanojia et al. [13]	120	21-40	-	-	-	13	-
Kashyap et al. [14]	1226	-	5	19	-	-	-
Kumari et al. [15]	400	18-45	-	-	8	-	-
Malhotra et al. [16]	555	20-40	15 (524)	46 (524)	-	132 (524)	-
Sharma et al. [17]	215	-	3 (151)	12 (151)	-	52 (151)	-
Naaz et al. [18]	50	20-37	3	0	2	-	2
Pathak et al. [19]	70	20-45	-	20	20	-	12
Prasad et al. [20]	150	18-40	2	5	1	22	-
Sankar et al. [21]	620	21-35	4	25	8	135	-
Saxena et al. [22]	62	-	-	3	4	3	3
Sethi et al. [23]	300	-	2	7	6	65	-
Sharma et al. [24]	137	20-41	4	-	-	80	31
Shrivastava and Jain [25]	50	-	4	2	4	7	-
Srivastava et al. [26]	218	-	1	12	3	84	-
Sinha et al. [27]	173	18-50	-	-	-	34	-
Thangappah et al. [28]	72	20-25	6	4	5	18 (49)	-
Thangappah and Narayanan [29]	173	20-37	8	6	7	45 (160)	
Tiwari et al. [30]	176	20-40	3	3	2	15	2

**Table 2:** Diagnostic accuracy of various tests for endometrial biopsy in detecting female genital tuberculosis ZN: Ziehl-Neelsen; HPE: histopathological examination; TB-PCR: tuberculosis-polymerase chain reaction; CRS: composite reference standard; SN: sensitivity in %; SP: specificity in %; NPV: negative predictive value in %; PPV: positive predictive value in %

Author	ZN staining				Culture				HPE				TB-PCR				GeneXpert				Reference test
	SN	SP	NPV	PPV	SN	SP	NPV	PPV	SN	SP	NPV	PPV	SN	SP	NPV	PPV	SN	SP	NPV	PPV	
Ashwini et al. [7]	-	-	-	-	-	-	-	-	-	-	-	-	34.78	99.08	99.13	33.13	6.90	99.79	98.16	40	Culture
Chaudhary et al. [9]	-	-	-	-	5.12	100	62.24	100	2.56	100	61.61	100	-	-	-	-	5.12	98.36	61.85	66.66	CRS
Chopra et al. [31]	1.42	100	-	-	8.57	100	-	-	21.42	100	-	-	72.85	100	-	-	-	-	-	-	CRS
Dogra et al. [10]	-	-	-	-	42.85	95	96	75	75	95.83	99	42.85	100	88.54	100	46.66	-	-	-	-	CRS
Gajbhiye et al. [11]	50	97.96	-	-	50	97.96	-	-	-	-	-	-	100	91.84	-	-	-	-	-	-	Culture
Kanade et al. [12]	14.28	100	-	-	-	-	-	-	-	-	-	-	-	-	-	-	66.67	100	-	-	Culture
Kanojia et al. [13]	-	-	-	-	-	100	-	-	-	95	-	-	-	-	-	-	-	-	-	-	Culture
Kanti et al. [32]	-	-	-	-	-	-	-	-	-	-	-	-	-	-	-	-	100	100	100	100	HPE
Paine et al. [33]	-	-	-	-	41.8	100	-	-	64.8	93.23	32.43	93.9	95.8	84.3	66.67	87.5	-	-	-	-	CRS
Saxena et al. [22]	-	-	-	-	100	100	100	100	75	98.28	75	99.28	100	96.61	60	100	100	100	100	100	CRS
Sethi et al. [23]	2.94	100	77.85	100	10.29	100	79.18	100	8.82	100	78.91	100	95.59	100	98.72	100	-	-	-	-	CRS
Sharma et al. [24]	4.60	100	49.08	100	-	-	-	-	-	-	-	-	-	-	-	-	35.63	100	100	58.82	CRS
Shrivastava and Jain [25]	8.33	100	94.9	100	-	-	-	-	-	16.7	99.5	95.3	-	-	-	-	-	-	-	-	Culture
Thangappah et al. [28]	-	-	-	-	7.14	100	-	-	10.7	100	-	-	57.1	90.5	-	-	-	-	-	-	Clinical criteria
Thangappah and Narayanan [29]	6.70	98.90	61.50	80	6.60	100	61.70	100	8.20	100	62.10	100	44.30	80.40	68.50	60	-	-	-	-	CRS
Tiwari et al. [30]	16.67	100	-	-	16.67	100	-	-	11.11	100	-	-	77.78	99.37	-	-	11.11	100	-	-	CRS

With regard to sensitivity and specificity, ZN staining exhibited low sensitivity, ranging from 1.42% to 50%, but demonstrated a consistent specificity of 100% across various studies. Culture demonstrated variable sensitivity, ranging from 5.12% to 50%, with high specificity (≥95%). HPE showed high specificity, as observed by Chaudhary et al., Chopra et al., Sethi et al., Thangappah et al., and Tiwari et al., and positive predictive value but a low negative predictive value, suggesting highly reliable performance in ruling out disease in negative cases [9,23,28,30,31]. The sensitivity values of HPE exhibited substantial variability, ranging from as low as 2.56% (Chaudhary et al.) to as high as 75%, as reported by Dogra et al. and Saxena et al. [9,10,22]. Apart from Chaudhary et al., much lower sensitivity values were observed in studies by Sethi et al., Thangappah et al., and Tiwari et al., reporting sensitivities of 8.8%, 8.2%, and 11.1%, respectively [9,23,29,30]. TB-PCR presented a wide sensitivity range from 34.78% to 100%, while specificity remained near 100% in most studies [10,11,23,31]. Several others, such as Saxena et al., Tiwari et al., and Ashwini et al., also demonstrated high specificity (>96%) [7,22,30]. GeneXpert demonstrated high specificity (98.36-100%) but widely varying sensitivity (5.1-100%). Saxena et al. reported 100% sensitivity and specificity of the culture method and GeneXpert, taking composite reference standards (CRS), which combine clinical, histopathological, and microbiological criteria to improve diagnostic accuracy, as the gold standard [22].

Table 3 presents the diagnostic performance of conventional and molecular methods for diagnosing FGTB across various other sample types, including peritoneal fluid/washings, menstrual blood, fallopian tube biopsy, placenta, urine, and vaginal discharge [8,11,14-17,27,29,32,34-38]. Most of the studies did not mention sensitivity or specificity data. For peritoneal fluid/washings, ZN staining and culture showed very low positivity, with only a few cases identified across studies. TB-PCR, however, showed higher specificity but exhibited low sensitivity (19.8% in the Thangappah and Narayanan study) [29]. In menstrual blood, TB-PCR demonstrated promising diagnostic potential, with sensitivity and specificity of 72.3% and 82.9%, respectively, in Chaubey et al. [8]. However, other methods performed on menstrual blood, including ZN staining and culture, showed low positivity rates across studies. Other non-conventional samples, such as fallopian tube biopsy, placental samples, urine, and vaginal discharge, were rarely investigated (only one study for each), and these samples yielded very few positive cases by conventional tests. In urine samples, TB-PCR demonstrated extremely low sensitivity (7.7% in Thangappah et al.), suggesting limited utility for non-invasive diagnosis [28]. Vaginal discharge also showed no positive cases across all diagnostic methods, indicating that this sample type may not be useful for FGTB diagnosis. In five studies, the investigator utilized multiple sample types for FGTB diagnosis [32,35-38]. Bhanothu et al. showed that TB-PCR targeting specific gene markers (e.g., 19 kDa, TRC4, MPB64, and 32 kDa) achieved high sensitivity (70.29-88.12%) and 100% specificity [36].

**Table 3:** Diagnostic accuracy of conventional and molecular methods for diagnosing female genital tuberculosis across various sample types n: sample size; ZN: Ziehl-Neelsen; HPE: histopathological examination; TB-PCR: tuberculosis-polymerase chain reaction; CRS: composite reference standard; SN: sensitivity in %; SP: specificity in %; NPV: negative predictive value in %; PPV: positive predictive value in %

Author	n	Age range (in years)	ZN staining			Culture			HPE			TB-PCR			GeneXpert			Reference test
			P	SN	SP	P	SN	SP	P	SN	SP	P	SN	SP	P	SN	SP	
Peritoneal fluid/washings																		
Agrawal et al. [34]	57	30.16+4.38	4	-	-	1	-	-	-	-	-	-	-	-	-	-	-	-
Sinha et al. [27]	173	18-50	-	-	-	-	-	-	-	-	-	42	-	-	-	-	-	-
Thangappah and Narayanan [29]	173	20-37	5	6.20	-	0	-	-	-	-	-	16	19.80	-	-	-	-	CSR
Menstrual blood																		
Chaubey et al. [8]	194	19-38	-	-	-	-	-	-	-	-	-	95	72.30	82.90	-	-	-	CRS
Gajbhiye et al. [11]	50	18-40	-	-	-	-	-	-	-	-	-	1	-	-	-	-	-	-
Kashyap et al. [14]	125	-	4	-	-	4	-	-	-	-	-	-	-	-	-	-	-	-
Kumari et al. [15]	400	18-45	8	-	-	13	-	-	-	-	-	51	-	-	-	-	-	-
Malhotra et al. [16]	555	20-40	0	-	-	0	-	-	-	-	-	2	-	-	-	-	-	-
Sharma et al. [17]	215	20-45	1	-	-	4	-	-	-	-	-	19	-	-	-	-	-	-
Fallopian tube biopsy																		
Malhotra et al. [16]	555	20-40	0	-	-	0	-	-	-	-	-	1	-	-	-	-	-	-
Placenta																		
Sharma et al. [17]	215	20-45	0	-	-	1	-	-	-	-	-	3	-	-	-	-	-	-
Urine																		
Thangappah and Narayanan [29]	173	20-37	-	-	-	0	-	-	-	-	-	4	7.70	-	-	-	-	CRS
Vaginal discharge																		
Malhotra et al. [16]	555	20-40	-	-	-	0	-	-	0	-	-	0	-	-	-	-	-	-
Multiple tissue type																		
Bhalerao and Sarma [35]	42	23-33	-	-	-	2	-	-	3	-	-	11	-	-	-	-	-	SN and SP are mentioned of each test as compared with other tests but not with CRS
Bhanothu et al. (endo-ovarian tissue biopsies and pelvic aspirated fluid) [36]	202	18-40	44	21.78	100	85	42.08	99	104	51.48	99	175 (19 kDa)	86.63	100	-	-	-	CRS
												175 (TRC4)	86.63	100				
												142 (MPB64)	70.29	100				
												178 (32 kDa)	88.12	100				
Farhana et al. [37]	87	21-50	-	-	-	4	-	-	-	-	-	-	-	-	7	-	-	-
Gurjar et al. [38]	100	20-35	-	-	-	28	-	-	23	60.71	52.77	52	64.28	91.6	-	-	-	Culture
Kanti et al. [32]	91	20-50	-	-	-		-	-	2	-	-	-	-	-	2	100	100	HPE

**Figure 2:**
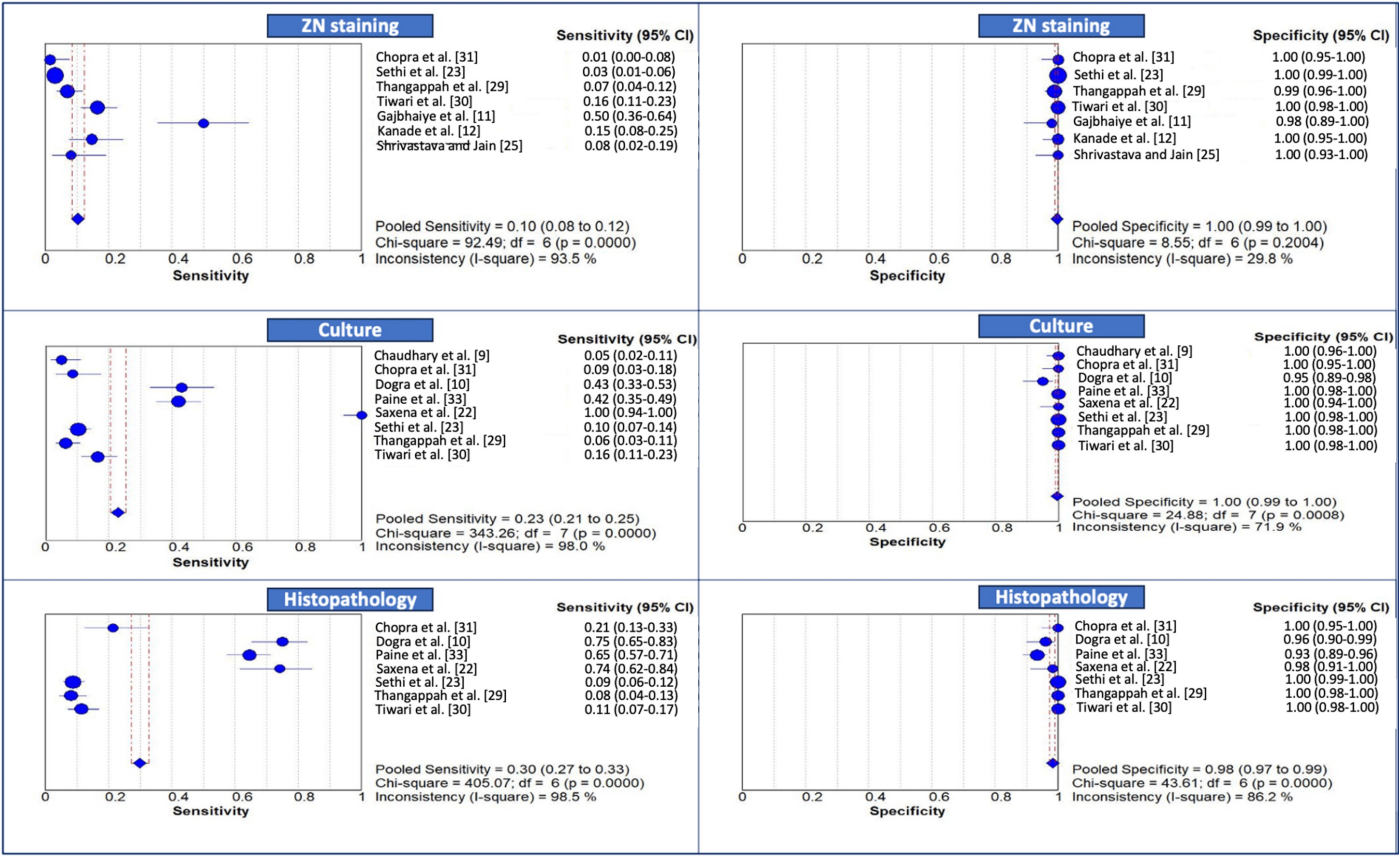
Forest plot of sensitivity and specificity of (a, b) ZN staining, (c, d) culture, and (e, f) histopathology for FGTB detection ZN: Ziehl-Neelsen; FGTB: female genital tuberculosis

The forest plot of sensitivity and specificity of the conventional methods, that is, ZN staining, culture, and HPE, for FGTB detection is presented in Figure 2. The pooled sensitivity of ZN staining, calculated using a random-effects model, was 0.10 (95% CI: 0.08-0.12), signifying that the test correctly identifies only 10% of the true-positive cases overall. Furthermore, the I^2^ statistic of 93.5%, the chi-squared value of 92.49, and a p-value of <0.0001 indicate statistically significant heterogeneity among the studies. On the other hand, its pooled specificity was 1.00 (95% CI: 0.99-1.00), underscoring the exceptional ability of the test to correctly identify true-negative cases. Furthermore, the I^2^ statistic was 29.8%, suggesting low to moderate heterogeneity among the included studies with regard to specificity. The pooled sensitivity estimate of culture was 0.23 (95% CI: 0.21-0.25), indicating that the test correctly identifies only 23% of true-positive cases on average. The chi-squared value of 343.26, the high I² value of 98%, and a p-value of <0.0001 indicate statistically significant heterogeneity across the included studies. Its pooled specificity was 1.00 (95% CI: 0.99-1.00), confirming that culture as a diagnostic tool for FGTB is highly accurate in identifying individuals who do not have the disease. However, despite the high pooled estimate, heterogeneity was noted among the studies, with an I² value of 71.9%. With regard to HPE, the pooled sensitivity was calculated as 0.30 (95% CI: 0.27-0.33) and the pooled specificity as 0.98 (95% CI: 0.97-0.99), with high I² values of 98.5% and 86.2%, highlighting marked heterogeneity among the studies.

**Figure 3:**
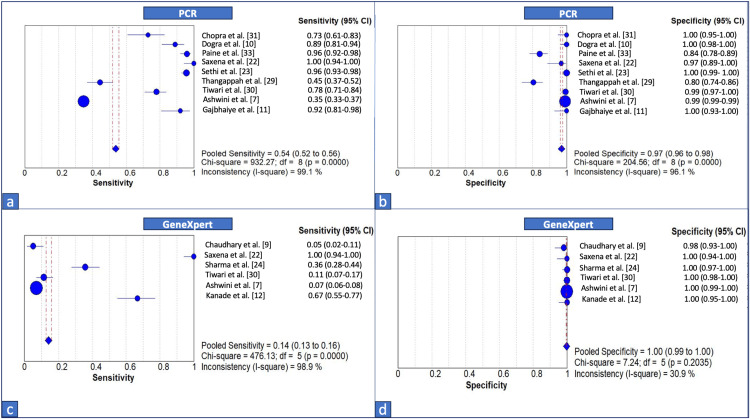
Forest plot of sensitivity and specificity of (a, b) PCR and (c, d) GeneXpert testing in detecting FGTB cases PCR: polymerase chain reaction; FGTB: female genital tuberculosis

Figure 3 illustrates the forest plot of sensitivity and specificity of PCR and GeneXpert testing in detecting FGTB cases. The pooled sensitivity for PCR was calculated to be 0.54 (95% CI: 0.52-0.56), suggesting that overall, PCR detects just over half of the true-positive cases. The pooled specificity was 0.97 (95% CI: 0.96-0.98), which still reflects the excellent overall diagnostic performance of PCR in terms of specificity. But, the I² statistic was high for both sensitivity and specificity, reflecting substantial differences across studies, potentially due to variations in PCR assay design, specimen type, technical protocols, disease prevalence, or patient selection criteria. For GeneXpert, the pooled sensitivity was just 0.14 (95% CI: 0.13-0.16), indicating that, on average, GeneXpert detected only 14% of true-positive cases. This is a concerningly low sensitivity, suggesting that GeneXpert, when used alone, may miss a substantial proportion of affected individuals. Furthermore, there was substantial heterogeneity among the studies, with a chi-squared value of 476.13 (df=5; p<0.0001) and an I² statistic of 98.9%. The pooled specificity of GeneXpert across all studies is 1.00 (95% CI: 0.99-1.00), which confirms that GeneXpert is highly reliable in correctly identifying individuals who do not have the disease. The low chi-squared value (7.24) and a non-significant p-value (0.2035) indicate that there is no statistically significant heterogeneity among the studies with respect to specificity. Further, an I² statistic of 30.9% suggests that the observed differences between study-specific estimates are likely due to random variation rather than true heterogeneity.

Among the included studies, one of the primary concerns was high patient selection bias, due to unclear recruitment methods, such as the lack of specification on whether random or consecutive sampling was used. Additionally, exclusion criteria (e.g., prior TB treatment, male factor infertility, incomplete diagnostic workups) introduced spectrum bias by selectively including certain patient subgroups. Furthermore, the use of clinical suspicion as an inclusion criterion adds subjectivity, impacting reproducibility. The index tests, particularly GeneXpert and PCR, frequently show unclear risks of bias due to the lack of blinded interpretation of results, raising concerns about interpretation bias. Many studies fail to report how indeterminate results are handled, which increases the risk of selective reporting bias. Additionally, positivity thresholds for molecular tests, such as PCR cycle cut-offs, are often not well defined, leading to inconsistencies in test interpretation. The reference standard, typically a CRS combining smear/culture for AFB, histopathology (epithelioid granulomas), and laparoscopic findings, also presented an unclear risk of bias. Some CRS components, particularly laparoscopic findings categorized as "probable" TB, introduced subjectivity and classification bias. Additionally, blinding of reference standard interpretation to index test results was rarely reported, increasing the risk of confirmation bias. Another concern was verification bias, as not all included studies used the same reference standard assessments, affecting the consistency of diagnostic accuracy estimates. A major issue across studies was flow and timing bias, frequently rated as high risk. Many studies failed to standardize the time intervals between the index test and reference standard, leading to concerns about disease progression bias. Additionally, some studies excluded patients with missing test results without clarifying how missing data were handled, leading to attrition bias.

**Figure 4:**
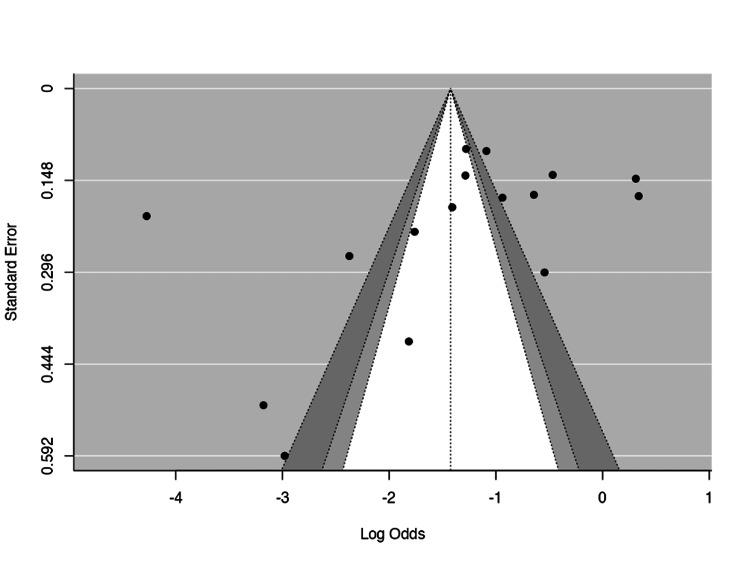
Funnel plot for publication bias

To assess potential publication bias in the included studies for meta-analysis [7,8,10,16,17,20-30], multiple methods were used. A funnel plot was generated, plotting the logit-transformed FGTB positivity rates (detected by PCR) against their standard error (Figure 4). Egger's regression test for funnel plot asymmetry showed a statistically significant result with a Z-value of -2.324 and a p-value of 0.020 (p<0.10), indicating the presence of small-study effects and potential publication bias. A higher p-value threshold (0.10) is used in this context due to the low sensitivity of these methods in small meta-analyses. Rosenthal's fail-safe N was calculated to be 3,951 (p<0.001), indicating that an additional 3,951 null-effect studies would be required to reduce the overall effect size to non-significance. This high value suggests that the findings of the meta-analysis are robust and unlikely to be substantially influenced by unpublished negative studies. Taken together, while the fail-safe N suggests that the findings are robust, the statistically significant result from Egger's test implies a possible presence of publication bias. Caution is therefore warranted in interpreting the pooled estimates, and the potential impact of small-study effects should be considered in the overall interpretation.

Discussion

Of the myriad diagnostic tools for diagnosing TB, ZN staining has been widely used due to its simplicity, affordability, and accessibility, particularly in low-resource settings. However, it is fraught with low sensitivity (~60-70%). Fluorescence microscopy, using auramine staining, improves sensitivity by ~10% compared to ZN staining but requires specialized equipment and darkroom conditions. The light-emitting diode (LED) fluorescence microscopy, endorsed by the World Health Organization (WHO), enhances diagnostic accuracy, is cost-effective, and has a longer lifespan, making it a practical alternative in resource-limited areas [39]. Culture-based methods remain the gold standard for TB detection, particularly for confirming cases missed by microscopy. Solid culture media, such as Löwenstein-Jensen (LJ) and Stonebrink medium, are highly specific and allow drug susceptibility testing (DST), but they require up to eight weeks for results. Liquid culture systems (e.g., MGIT, BACTEC) offer higher sensitivity (~20% more than solid culture) and faster turnaround times (10-14 days), though they are expensive and susceptible to contamination.

Molecular diagnostic techniques provide a rapid and more accurate alternative to traditional methods. Nucleic acid amplification tests (NAATs), such as PCR-based methods, detect *Mycobacterium tuberculosis* DNA with high specificity, yielding results within 3-6 hours. However, their sensitivity varies, especially in smear-negative samples, and they require skilled personnel. The GeneXpert MTB/RIF (Cepheid, Sunnyvale, California, United States), an automated assay, can diagnose TB and rifampicin resistance in about two hours, making it a cornerstone of modern TB diagnostics. Truenat MTB (Molbio, Verna, Goa, India), a point-of-care test, is particularly useful in resource-limited settings, demonstrating comparable sensitivity to GeneXpert [40]. The WHO recommends GeneXpert MTB/RIF and Truenat MTB for diagnosing both pulmonary and extrapulmonary TB [41,42]. Loop-mediated isothermal amplification (LAMP) is a faster and more affordable molecular alternative that does not require complex equipment, but its sensitivity remains lower than GeneXpert.

While GeneXpert remains widely adopted for its simplicity and rapid turnaround time, newer technologies such as Xpert Ultra, droplet digital PCR (ddPCR), and metagenomic next-generation sequencing (mNGS) offer superior sensitivity. However, these advanced methods may require additional resources and expertise, necessitating careful selection based on clinical and logistical considerations. GeneXpert MTB/RIF Ultra (Xpert Ultra) improves upon the original GeneXpert MTB/RIF, with a lower detection limit (16 cfu/mL vs. 114 cfu/mL) and the inclusion of multicopy amplification targets IS6110 and IS1081, along with the rifampicin resistance-determining region (RRDR) of the rpoB gene [43]. ddPCR has demonstrated higher sensitivity (99%) compared to GeneXpert (64%), making it an effective tool for detecting low mycobacterial DNA levels. It also exhibits high specificity (96.6%) and does not require a standard curve for result interpretation [44,45]. mNGS can offer higher sensitivity (86%) and specificity (100%) than GeneXpert but is more complex and requires advanced bioinformatics capabilities [44]. Using this technique, the genes responsible for drug resistance, such as rpoB, embB, pncA, rpsA, gyrA, gyrB, rrs, and eis, can be amplified. This method is highly sensitive for detecting mutations associated with resistance to isoniazid, rifampicin, streptomycin, ofloxacin, levofloxacin, and moxifloxacin [46].

Our meta-analysis revealed that the ZN staining demonstrates poor sensitivity for FGTB detection with substantial heterogeneity among studies. But, it showed a consistently high specificity across multiple studies, with a pooled estimate of 1.00, with low heterogeneity among the included studies. Clinically, this implies that the test is likely to miss a large proportion of true-positive cases but is highly effective in excluding the disease in non-affected individuals, thereby minimizing the risk of false positives. The forest plot of culture as a diagnostic modality revealed that while the culture method demonstrates excellent specificity, it may occasionally demonstrate high sensitivity in isolated studies, and its overall pooled sensitivity remains quite low, with significant inter-study variability. Our findings highlight the limitations of traditional diagnostic methods such as ZN staining and culture techniques, which have demonstrated suboptimal sensitivity in detecting *Mycobacterium tuberculosis* in genital tract samples. ZN staining, although specific, suffers from poor sensitivity due to the low bacillary load in FGTB cases [1]. Similarly, culture, despite being a gold standard in pulmonary TB, often yields inconclusive results due to the difficulty in obtaining adequate specimens from deep-seated genital organs and the prolonged time required for mycobacterial growth [1,4]. HPE has shown a variable diagnostic yield, with granulomatous inflammation serving as an important but nonspecific indicator of TB. While histopathology provides supportive evidence for FGTB, it cannot serve as a standalone diagnostic modality due to the overlap with other granulomatous conditions. Molecular methods, including PCR and GeneXpert MTB/RIF, have emerged as promising alternatives. The presence of considerable heterogeneity in the included studies, potentially due to variations in PCR assay design, technical protocols, disease prevalence, or patient selection criteria, however, suggests that results should be interpreted cautiously and in context with study-specific conditions. GeneXpert demonstrates near-perfect pooled specificity, making it a highly accurate tool for ruling out the disease. The minimal variability across studies strengthens confidence in its consistent performance in correctly identifying true negatives, which is a critical aspect in avoiding unnecessary treatment and anxiety among individuals who are disease-free. But given its low pooled sensitivity, GeneXpert should not be used as a standalone test for diagnosis in this context and may require complementary testing methods to improve diagnostic accuracy.

Our review also indicates that while these molecular techniques offer superior sensitivity compared to conventional methods, their diagnostic accuracy varies depending on the specimen type. The choice of specimen type plays a crucial role in diagnostic accuracy. Endometrial biopsy and aspirate samples are commonly used due to their accessibility, as evident in this review. Menstrual blood also showed promise as a non-invasive sample with relatively high TB-PCR sensitivity. However, conventional methods such as ZN staining and culture exhibited limited diagnostic value due to their low sensitivity. Pelvic washings and peritoneal fluid analysis have shown potential, but their role in routine diagnosis needs further validation. Tubo-ovarian biopsy, placental tissue, urine, and vaginal discharge demonstrated lower diagnostic yields and were found to be the least useful sample types. Hence, our findings highlight the need for molecular techniques in improving the diagnosis of FGTB. Also, the findings suggest that a combination approach utilizing multiple specimen types may enhance diagnostic sensitivity, especially in cases where endometrial involvement is uncertain. Altez-Fernandez et al. conducted a systematic review on the accuracy of NAATs in urine for diagnosing genitourinary TB [47]. PCR-based NAATs were highly heterogeneous, lacked standardization, and had quality concerns, preventing pooled analysis. A single study assessed ligase chain reaction (LCR), limiting conclusions [47]. In contrast, GeneXpert MTB/RIF showed good quality and consistency, with a pooled sensitivity of 87% (95% CI: 66-96%) and specificity of 91% (95% CI: 84-95%). While GeneXpert MTB/RIF appears promising, further high-quality studies are needed to refine accuracy estimates and evaluate its role in detecting drug-resistant genitourinary TB.

Apart from these tests, serological and antigen-based tests have also been explored for TB diagnosis. Serodiagnostic (antibody-based) tests are simple and low cost but have poor sensitivity and specificity, leading the WHO to advise against their use. Emerging methods such as immuno-PCR and aptamer-based assays show promise in improving detection accuracy, particularly for extrapulmonary TB, but they remain in the research phase and are costly. Immuno-PCR combines immunoassay principles with PCR amplification, enhancing sensitivity and specificity, whereas aptamer-based assays use single-stranded DNA or RNA molecules that bind specifically to target molecules. Sun et al. developed a universal enzyme-linked immunosorbent assay (ELISA) for TB detection across multiple hosts. This test utilizes a fusion protein (MMEC) comprising MPB70, MPB83, ESAT6, and CFP10 as a coating antigen, making it applicable across various mycobacterial species. The ELISA demonstrated 100% sensitivity and 94.85% specificity for detecting TB in humans, proving to be a reliable diagnostic method [48]. Lipoarabinomannan (LAM) detection in urine has been introduced as a rapid diagnostic tool, particularly for immunocompromised patients with low CD4 counts. While it is beneficial in human immunodeficiency virus (HIV)-TB co-infected individuals, its sensitivity in non-HIV patients is limited.

Detecting latent TB remains a significant challenge. The tuberculin skin test (TST/Mantoux test) is commonly used due to its affordability and simplicity. However, it cannot differentiate between latent and active TB and is influenced by prior Bacillus Calmette-Guérin (BCG) vaccination [49]. Interferon-gamma release assays (IGRAs), such as QuantiFERON-TB Gold, offer greater specificity and are unaffected by BCG vaccination, making them a superior alternative. However, they are costly and require specialized laboratory infrastructure. QuantiFERON-TB Gold detects TB by measuring interferon-gamma (IFN-γ) production in response to mycobacterial antigens (ESAT-6 and CFP-10) in a blood sample [45,50]. Another IGRA, the T-SPOT.TB/enzyme-linked immunospot (ELISPOT) assay, identifies TB infection by quantifying cytokine-producing T cells [45].

DST is crucial for diagnosing multidrug-resistant TB (MDR-TB) [51]. Culture-based DST remains the gold standard, providing definitive resistance profiles but requiring 4-8 weeks for results. More advanced molecular methods, such as line probe assays and whole-genome sequencing, allow for the rapid detection of drug resistance mutations within 48 hours [52]. While these methods provide early resistance detection, they are limited to known mutations and require sophisticated facilities.

Recent advancements in TB diagnostics include nanoparticle-based methods (quantum dot detection systems, magnetic nanoparticles, and biosensors), CRISPR-Cas technology, and microRNA-based assays, all of which exhibit excellent sensitivity and specificity and form the basis for a rapid point-of-care diagnostic tool for TB detection [45]. Mass spectrometry-based methods, such as matrix-assisted laser desorption/ionization-time of flight (MALDI-TOF), enable the species-level identification of *Mycobacterium tuberculosis* complex (MTBC) and can detect antibiotic resistance through protein and lipid profile analysis, aiding in the identification of drug-resistant TB strains [53].

This systematic review and meta-analysis possesses several key strengths. It comprehensively evaluates the diagnostic performance of various pelvic-derived samples, providing valuable insights into the most effective specimen types for detecting FGTB. By assessing both conventional methods (ZN staining, culture, HPE) and molecular techniques (PCR and GeneXpert), the study offers a robust comparative framework that reflects current diagnostic practices across diverse clinical settings. Given that FGTB is often underdiagnosed due to its paucibacillary nature and nonspecific clinical presentation, this review addresses a significant gap in the literature and contributes toward improved diagnostic accuracy. The inclusion of a range of sample types enhances the clinical applicability of the findings and may inform optimal specimen selection to improve diagnostic yield. However, there are several limitations to consider, such as heterogeneity between studies, small sample sizes, and variable reference standards used. Also, regarding publication bias assessment, although the fail-safe N indicates that the results are likely to be stable, the statistically significant Egger's test suggests a potential publication bias; hence, these results have to be interpreted with caution, as these statistical approaches are known to have low power and yield unreliable results in small meta-analyses [54]. Although formal quantitative assessment was not feasible, the possibility of publication bias cannot be completely ruled out and is acknowledged as a limitation of this review. Despite these limitations, this review addresses an important evidence gap in the diagnostic landscape of FGTB and provides direction for both clinical decision-making and future research.

## Conclusions

Our systematic review underscores the diagnostic complexities associated with FGTB and highlights the need for an integrated approach incorporating molecular, microbiological, and histopathological methods. Based on our findings, we recommend endometrial biopsy/aspirate to be the optimal sample type for FGTB diagnosis. Given the variable sensitivity and specificity of different tests and specimen types, clinicians should adopt a multi-modality approach to improve diagnostic accuracy. Molecular assays such as GeneXpert MTB/RIF should be integrated into the diagnostic workflow, particularly for high-risk patients, while histopathology and culture can provide complementary information. However, the reliance on a single test should be avoided due to inherent limitations. Future research should focus on standardizing sample collection and processing techniques to optimize diagnostic performance. Large-scale prospective studies are required to validate the efficacy of newer molecular assays and determine their role in routine clinical practice. Additionally, efforts should be made to improve accessibility to advanced diagnostic tools in resource-limited settings where FGTB is prevalent.

## Appendices

Appendix 1

**Table 4:** Complete search strategy

Search strategy		
Disease concept block	MeSH Terms for Disease "Tuberculosis, Female Genital"[Mesh] "Tuberculosis, Urogenital"[Mesh] AND "Female"[Mesh] "Tuberculosis"[Mesh] AND "Genital Diseases, Female"[Mesh]	Combined Disease Concept #1 OR #2 OR #3 OR #4 OR #5
	Text Words for Disease (female genital tuberculosis[Title/Abstract]) OR (genital TB[Title/Abstract]) OR (genital tuberculosis[Title/Abstract] AND female[Title/Abstract]) OR (pelvic tuberculosis[Title/Abstract] AND female[Title/Abstract]) OR (reproductive tract tuberculosis[Title/Abstract] AND female[Title/Abstract]) ((tuberculosis[Title/Abstract]) AND (genital tract[Title/Abstract] OR endometri*[Title/Abstract] OR tubal[Title/Abstract] OR fallopian[Title/Abstract] OR ovari*[Title/Abstract] OR cervix[Title/Abstract] OR cervical[Title/Abstract] OR uterine[Title/Abstract] OR vaginal[Title/Abstract] OR vulvar[Title/Abstract] OR pelvic[Title/Abstract])) AND female[Title/Abstract]	
Diagnostic test concept block	MeSH Terms for Diagnostic Tests "Diagnosis"[Mesh] OR "Diagnostic Techniques and Procedures"[Mesh] OR "Clinical Laboratory Techniques"[Mesh] "Sensitivity and Specificity"[Mesh] OR "Predictive Value of Tests"[Mesh] OR "ROC Curve"[Mesh] OR "Diagnostic Errors"[Mesh] "Microscopy"[Mesh] OR "Mycobacterium tuberculosis/isolation and purification"[Mesh] OR "Culture Techniques"[Mesh] "Polymerase Chain Reaction"[Mesh] OR "Nucleic Acid Amplification Techniques"[Mesh] OR "In Situ Hybridization"[Mesh] "Histological Techniques"[Mesh] OR "Biopsy"[Mesh] OR "Pathology"[Mesh]	Combined Diagnostic Tests Concept #7 OR #8 OR #9 OR #10 OR #11 OR #12 OR #13 OR #14 OR #15 OR #16
	Text Words for Diagnostic Tests (diagnos*[Title/Abstract]) OR (detect*[Title/Abstract]) OR (identification[Title/Abstract]) OR (screening[Title/Abstract]) (sensitivity[Title/Abstract]) OR (specificity[Title/Abstract]) OR (ROC[Title/Abstract]) OR (predictive value[Title/Abstract]) OR (accuracy[Title/Abstract]) OR (yield[Title/Abstract]) (microscopy[Title/Abstract]) OR (AFB[Title/Abstract]) OR (acid fast bacilli[Title/Abstract]) OR (ZN stain[Title/Abstract]) OR (culture[Title/Abstract]) OR (MGIT[Title/Abstract]) OR (Lowenstein-Jensen[Title/Abstract]) OR (LJ medium[Title/Abstract]) (PCR[Title/Abstract]) OR (polymerase chain reaction[Title/Abstract]) OR (nucleic acid amplification[Title/Abstract]) OR (NAA[Title/Abstract]) OR (NAAT[Title/Abstract]) OR (GeneXpert[Title/Abstract]) OR (Xpert MTB[Title/Abstract]) OR (CBNAAT[Title/Abstract]) OR (molecular[Title/Abstract]) OR (TB-PCR[Title/Abstract]) OR (IS6110[Title/Abstract]) OR (LAMP[Title/Abstract]) OR (loop-mediated isothermal amplification[Title/Abstract]) (histopathology[Title/Abstract]) OR (histology[Title/Abstract]) OR (biopsy[Title/Abstract]) OR (histological[Title/Abstract]) OR (granuloma[Title/Abstract]) OR (pathology[Title/Abstract])	
Specimen type concept block	MeSH Terms for Specimen "Specimen Handling"[Mesh] OR "Diagnostic Techniques, Obstetrical and Gynecological"[Mesh] "Endometrium/microbiology"[Mesh] OR "Endometrium/pathology"[Mesh] "Peritoneal Fluid/microbiology"[Mesh] OR "Peritoneal Fluid/cytology"[Mesh] "Fallopian Tubes/microbiology"[Mesh] OR "Fallopian Tubes/pathology"[Mesh] "Ovary/microbiology"[Mesh] OR "Ovary/pathology"[Mesh] "Uterine Cervix/microbiology"[Mesh] OR "Uterine Cervix/pathology"[Mesh] "Menstruation/microbiology"[Mesh]	Combined Specimen Concept #18 OR #19 OR #20 OR #21 OR #22 OR #23 OR #24 OR #25 OR #26 OR #27 OR #28 OR #29 OR #30 OR #31
	Text Words for Specimen (specimen[Title/Abstract]) OR (sample[Title/Abstract]) OR (tissue[Title/Abstract]) OR (aspirate[Title/Abstract]) OR (biopsy material[Title/Abstract]) OR (washing[Title/Abstract]) OR (fluid[Title/Abstract]) (endometri*[Title/Abstract]) OR (endometrial biopsy[Title/Abstract]) OR (endometrial curettage[Title/Abstract]) OR (endometrial aspirate[Title/Abstract]) OR (uterine curettage[Title/Abstract]) OR (D&C[Title/Abstract]) (peritoneal fluid[Title/Abstract]) OR (pelvic fluid[Title/Abstract]) OR (peritoneal washing[Title/Abstract]) OR (pouch of Douglas[Title/Abstract]) OR (cul-de-sac fluid[Title/Abstract]) (fallopian tube[Title/Abstract]) OR (tubal[Title/Abstract]) OR (salpingeal[Title/Abstract]) (ovarian[Title/Abstract]) OR (ovary[Title/Abstract]) OR (ovarian biopsy[Title/Abstract]) (cervical[Title/Abstract]) OR (cervix[Title/Abstract]) OR (cervical smear[Title/Abstract]) OR (cervical aspirate[Title/Abstract]) OR (cervical biopsy[Title/Abstract]) (menstrual blood[Title/Abstract]) OR (menstrual fluid[Title/Abstract]) OR (menstrual discharge[Title/Abstract]) OR (menstrual sample[Title/Abstract])	
Complete Search Strategy #6 AND #17 AND #32 No filters used		

Appendix 2

**Table 5:** Checklist of PRISMA guidelines PRISMA: Preferred Reporting Items for Systematic Reviews and Meta-Analyses; PROSPERO: International Prospective Register of Systematic Reviews

Section and topic	Item #	Checklist item	Location where the item is reported
Title			
Title	1	Identify the report as a systematic review.	Title
Abstract			
Abstract	2	See the PRISMA 2020 for Abstracts checklist.	Seen and implemented
Introduction			
Rationale	3	Describe the rationale for the review in the context of existing knowledge.	Described in the Introduction section
Objectives	4	Provide an explicit statement of the objective(s) or question(s) the review addresses.	Mentioned in the Introduction section
Methods			
Eligibility criteria	5	Specify the inclusion and exclusion criteria for the review and how studies were grouped for the syntheses.	Described in the Methodology section
Information sources	6	Specify all databases, registers, websites, organizations, reference lists, and other sources searched or consulted to identify studies. Specify the date when each source was last searched or consulted.	Done in the Methodology section and PRISMA diagram
Search strategy	7	Present the full search strategies for all databases, registers, and websites, including any filters and limits used.	Appendix 1
Selection process	8	Specify the methods used to decide whether a study met the inclusion criteria of the review, including how many reviewers screened each record and each report retrieved, whether they worked independently, and, if applicable, the details of automation tools used in the process.	Done in the Methodology section
Data collection process	9	Specify the methods used to collect data from reports, including how many reviewers collected data from each report, whether they worked independently, any processes for obtaining or confirming data from study investigators, and, if applicable, the details of automation tools used in the process.	Page 4
Data items	10a	List and define all outcomes for which data were sought. Specify whether all results that were compatible with each outcome domain in each study were sought (e.g., for all measures, time points, analyses) and, if not, the methods used to decide which results to collect.	Done in the Methodology section
	10b	List and define all other variables for which data were sought (e.g., participant and intervention characteristics, funding sources). Describe any assumptions made about any missing or unclear information.	Done in the Methodology section
Study risk of bias assessment	11	Specify the methods used to assess risk of bias in the included studies, including details of the tool(s) used, how many reviewers assessed each study and whether they worked independently, and, if applicable, the details of automation tools used in the process.	Done in the Methodology section
Effect measures	12	Specify for each outcome the effect measure(s) (e.g., risk ratio, mean difference) used in the synthesis or presentation of results.	Done in the Methodology section
Synthesis methods	13a	Describe the processes used to decide which studies were eligible for each synthesis (e.g., tabulating the study intervention characteristics and comparing against the planned groups for each synthesis (item #5)).	Tables 1-3
	13b	Describe any methods required to prepare the data for presentation or synthesis, such as handling of missing summary statistics or data conversions.	Supplementary tables/figures
	13c	Describe any methods used to tabulate or visually display the results of individual studies and syntheses.	Figures
	13d	Describe any methods used to synthesize results and provide a rationale for the choice(s). If meta-analysis was performed, describe the model(s), method(s) to identify the presence and extent of statistical heterogeneity, and software package(s) used.	Done in the Methodology section
	13e	Describe any methods used to explore possible causes of heterogeneity among study results (e.g., subgroup analysis, meta-regression).	Not done
	13f	Describe any sensitivity analyses conducted to assess the robustness of the synthesized results.	Not done
Reporting bias assessment	14	Describe any methods used to assess the risk of bias due to missing results in a synthesis (arising from reporting biases).	By searching the grey literature
Certainty assessment	15	Describe any methods used to assess certainty (or confidence) in the body of evidence for an outcome.	Not done
Results			
Study selection	16a	Describe the results of the search and selection process, from the number of records identified in the search to the number of studies included in the review, ideally using a flow diagram.	PRISMA Figure 1
	16b	Cite studies that might appear to meet the inclusion criteria, but which were excluded, and explain why they were excluded.	PRISMA Figure 1
Study characteristics	17	Cite each included study and present its characteristics.	Tables 1-3
Risk of bias in studies	18	Present assessments of risk of bias for each included study.	Discussed
Results of individual studies	19	For all outcomes, present, for each study, (a) summary statistics for each group (where appropriate) and (b) an effect estimate and its precision (e.g., confidence/credible interval), ideally using structured tables or plots.	Figure provided
Results of syntheses	20a	For each synthesis, briefly summarize the characteristics and risk of bias among contributing studies.	Tables/figures
	20b	Present the results of all statistical syntheses conducted. If meta-analysis was done, present for each the summary estimate and its precision (e.g., confidence/credible interval) and measures of statistical heterogeneity. If comparing groups, describe the direction of the effect.	Figures and results
	20c	Present the results of all investigations of possible causes of heterogeneity among study results.	Discussed
	20d	Present the results of all sensitivity analyses conducted to assess the robustness of the synthesized results.	Not done
Reporting biases	21	Present the assessments of risk of bias due to missing results (arising from reporting biases) for each synthesis assessed.	Not done
Certainty of evidence	22	Present the assessments of certainty (or confidence) in the body of evidence for each outcome assessed.	Not done
Discussion			
Discussion	23a	Provide a general interpretation of the results in the context of other evidence.	Done in the Results and Discussion section
	23b	Discuss any limitations of the evidence included in the review.	Discussed
	23c	Discuss any limitations of the review processes used.	Discussed
	23d	Discuss the implications of the results for practice, policy, and future research.	Discussed
Other information			
Registration and protocol	24a	Provide the registration information for the review, including the register name and registration number, or state that the review was not registered.	Provided in the Methodology section
	24b	Indicate where the review protocol can be accessed, or state that a protocol was not prepared.	On PROSPERO
	24c	Describe and explain any amendments to the information provided at registration or in the protocol.	Not applicable
Support	25	Describe the sources of financial or non-financial support for the review and the role of the funders or sponsors in the review.	Mentioned
Competing interests	26	Declare any competing interests of review authors.	Mentioned
Availability of data, code, and other materials	27	Report which of the following are publicly available and where they can be found: template data collection forms, data extracted from included studies, data used for all analyses, analytic code, and any other materials used in the review.	Supplementary material and available from the authors upon request

## References

[1] Sethi A, Bajaj B, Nair D, Pachauri D, Gupta M, Mahajan A (2022). Comparison of conventional methods with newer diagnostic modalities to detect genital tuberculosis in infertile women. J Obstet Gynaecol India.

[2] Grace GA, Devaleenal DB, Natrajan M (2017). Genital tuberculosis in females. Indian J Med Res.

[3] Vijay A, Tiwari N, Sharma A, Pandey G (2023). Correlation of female genital tuberculosis and infertility: a comprehensive systematic review, meta-analysis, and female genital tuberculosis infertility pathway analysis. J Midlife Health.

[4] Mishra D, Turuk J, Palo S, Ray PK, Mishra B, Pati S (2024). Peritoneal fluid from pouch of Douglas is not a suitable specimen for molecular testing in the diagnosis of female genital tuberculosis in women presenting with infertility. J Indian Med Assoc.

[5] Zamora J, Abraira V, Muriel A, Khan K, Coomarasamy A (2006). Meta-DiSc: a software for meta-analysis of test accuracy data. BMC Med Res Methodol.

[6] (2022). jamovi. https://www.jamovi.org/.

[7] Ashwini M, Ashwini N, Arunkumar N, Gunasheela D (2020). A study on diagnostic evaluation of two different rapid DNA polymerase chain reaction techniques namely Gene Xpert Mycobacterium tuberculosis/rifampin (MTB/RIF) and mycoreal polymerase chain reaction in the diagnosis of endometrial tuberculosis considering culture as gold standard. J Hum Reprod Sci.

[8] Chaubey L, Kumar D, Prakash V, Nath G (2019). Menstrual blood versus endometrial biopsy in detection of genital tuberculosis by using nested polymerase chain reaction in an endemic region. J Hum Reprod Sci.

[9] Chaudhary R, Dhama V, Singh M, Singh S (2020). Comparison of diagnostic accuracy of BACTEC culture, gene-xpert and histopathology in the diagnosis of genital tuberculosis in women with infertility. Int J Reprod Contracept Obstet Gynecol.

[10] Dogra A, Kaul I, Kanta S (2019). Endometrial tuberculosis pick by PCR; in female infertility. JK Science.

[11] Gajbhiye SB, Bhakre JB, Damle AS (2019). Assessment of endometrial specimens for tubercular infection among infertile women using DNA-PCR. J Clin Diagn Res.

[12] Kanade S, Solanki M, Thombare S, Nataraj G (2023). Utility of laboratory diagnostic tests in women suspected of genital tuberculosis attending a tertiary care teaching hospital. Int J Mycobacteriol.

[13] Kanojia S, Singh H, Gwal R, Agarwal S, Mishra V (2015). Evaluation of nested polymerase chain reaction technique in the diagnosis of female genital TB in endometrial samples. J Pure Appl Microbiol.

[14] Kashyap B, Kaur T, Jhamb R, Kaur IR (2012). Evaluating the utility of menstrual blood versus endometrial biopsy as a clinical sample in the diagnosis of female genital tuberculosis. Asian J Med Res.

[15] Kumari G, Goel D, Tiwari A, Mishra S (2017). Study on conventional and molecular diagnostic tools for genital tuberculosis associating with infertility in Indian women. Sci Int.

[16] Malhotra B, Sinha P, Hooja S, Vyas L (2012). Rapid diagnosis of genital tuberculosis by real-time polymerase chain reaction. J South Asian Feder Obst Gynae.

[17] Sharma N, Sharma V, Singh PR (2013). Diagnostic value of PCR in genitourinary tuberculosis. Indian J Clin Biochem.

[18] Naaz A, Sarbhai V, Sarbhai V (2021). Role of GeneXpert in endometrial biopsy for diagnosis of genital tuberculosis in women presenting with infertility. Int J Reprod Contracept Obstet Gynecol.

[19] Pathak A, Yadav D, Verma U, Gautam Y (2020). A study to compare the diagnostic accuracy of GeneXpert MTB/RIF/assay and its comparison with liquid culture in clinically suspected cases of genital tuberculosis attending outpatient department of tertiary center. Int J Reprod Contracept Obstet Gynecol.

[20] Prasad S, Singhal M, Negi SS, Gupta S, Singh S, Rawat DS, Rai A (2012). Targeted detection of 65 kDa heat shock protein gene in endometrial biopsies for reliable diagnosis of genital tuberculosis. Eur J Obstet Gynecol Reprod Biol.

[21] Sankar MM, Kumar P, Munawwar A (2013). Usefulness of multiplex PCR in the diagnosis of genital tuberculosis in females with infertility. Eur J Clin Microbiol Infect Dis.

[22] Saxena R, Shrinet K, Rai SN (2022). Diagnosis of genital tuberculosis in infertile women by using the composite reference standard. Dis Markers.

[23] Sethi S, Dhaliwal L, Dey P, Kaur H, Yadav R, Sethi S (2016). Loop-mediated isothermal amplification assay for detection of Mycobacterium tuberculosis complex in infertile women. Indian J Med Microbiol.

[24] Sharma JB, Dharmendra S, Jain S (2020). Evaluation of Gene Xpert as compared to conventional methods in diagnosis of female genital tuberculosis. Eur J Obstet Gynecol Reprod Biol.

[25] Shrivastava D, Jain J (2021). Comparison of laparoscopic findings with tuberculosis polymerase chain reaction in the diagnosis of genital tuberculosis among subfertile women. Journal of Datta Meghe Institute of Medical Sciences University.

[26] Srivastava I, Bhatambare GS, Deshmukh AB (2014). Genital tuberculosis: evaluating microscopy, culture, histopathology and PCR for diagnosis: all play their role. Int J Curr Microbiol App Sci.

[27] Sinha P, Malhotra B, Dawra R (2019). Evaluation of diagnostic value of pouch of Douglas fluid in comparison to endometrial biopsy samples for the diagnosis of genital tuberculosis by real-time polymerase chain reaction. J South Asian Feder Obst Gynae.

[28] Thangappah RB, Paramasivan CN, Narayanan S (2011). Evaluating PCR, culture & histopathology in the diagnosis of female genital tuberculosis. Indian J Med Res.

[29] Thangappah RB, Narayanan S (2018). Diagnosing genital tuberculosis in female infertility by clinical, histopathological, culture and polymerase chain reaction techniques: an evaluative study. Int J Reprod Contracept Obstet Gynecol.

[30] Tiwari K, Prasad S, Tanwar R (2020). Role of Gene Xpert in the detection of genital tuberculosis in endometrial tissue among women with infertility. J Hum Reprod Sci.

[31] Chopra S, Sharma S, Sharma K (2019). Evaluation of multiplex PCR for rapid diagnosis of female genital tuberculosis. J Assoc Physicians India.

[32] Kanti V, Seth S, Gupta S, Verma V, Singh A, Maurya G, Kumar A (2021). Comparison of the efficacy of the cartridge-based nucleic amplification (CBNAAT)/Xpert test and histology of genital tissues in diagnosing female genital tuberculosis. Cureus.

[33] Paine SK, Basu A, Choudhury RG, Bhattacharya B, Chatterjee S, Bhattacharya C (2018). Multiplex PCR from menstrual blood: a non-invasive cost-effective approach to reduce diagnostic dilemma for genital tuberculosis. Mol Diagn Ther.

[34] Agrawal S, Kant S, Das V, Jain A, Mishra S (2021). Limited role of GeneXpert in peritoneal fluid in the diagnosis of genital tuberculosis in infertile women. J Family Med Prim Care.

[35] Bhalerao A, Sarma S (2020). Evaluation of culture, polymerase chain reaction (PCR) and histopathology for diagnosis of genital tuberculosis in female infertility. Panacea J Med Sci.

[36] Bhanothu V, Theophilus JP, Rozati R (2014). Use of endo-ovarian tissue biopsy and pelvic aspirated fluid for the diagnosis of female genital tuberculosis by conventional versus molecular methods. PLoS One.

[37] Farhana A, Zahoor D, Manzoor M, Kanth F (2018). Evaluation of Xpert MTB/ RIF assay for the detection of female genital tuberculosis in a tertiary care center- a descriptive cross-sectional study. Microbiol Res J Int.

[38] Gurjar K, Meena KL, Rajoria L, Sharma N (2018). Comparison of diagnostic efficacy of USG, tuberculin test, nucleic acid amplification test (PCR) & histopathology for diagnosis of genital tuberculosis in infertile women, assuming culture as gold standard. IMJ Health.

[39] Chang EW, Page AL, Bonnet M (2016). Light-emitting diode fluorescence microscopy for tuberculosis diagnosis: a meta-analysis. Eur Respir J.

[40] Singh UB, Singh M, Sharma S (2023). Expedited diagnosis of pediatric tuberculosis using Truenat MTB-Rif Dx and GeneXpert MTB/RIF. Sci Rep.

[41] Penn-Nicholson A, Gomathi SN, Ugarte-Gil C (2021). A prospective multicentre diagnostic accuracy study for the Truenat tuberculosis assays. Eur Respir J.

[42] Hong JM, Lee H, Menon NV, Lim CT, Lee LP, Ong CW (2022). Point-of-care diagnostic tests for tuberculosis disease. Sci Transl Med.

[43] Osei Sekyere J, Maphalala N, Malinga LA, Mbelle NM, Maningi NE (2019). A comparative evaluation of the new Genexpert MTB/RIF Ultra and other rapid diagnostic assays for detecting tuberculosis in pulmonary and extra pulmonary specimens. Sci Rep.

[44] Zhang D, Yu F, Han D (2023). ddPCR provides a sensitive test compared with GeneXpert MTB/RIF and mNGS for suspected Mycobacterium tuberculosis infection. Front Cell Infect Microbiol.

[45] Mukherjee S, Perveen S, Negi A, Sharma R (2023). Evolution of tuberculosis diagnostics: from molecular strategies to nanodiagnostics. Tuberculosis (Edinb).

[46] Hillemann D, Weizenegger M, Kubica T, Richter E, Niemann S (2005). Use of the genotype MTBDR assay for rapid detection of rifampin and isoniazid resistance in Mycobacterium tuberculosis complex isolates. J Clin Microbiol.

[47] Altez-Fernandez C, Ortiz V, Mirzazadeh M, Zegarra L, Seas C, Ugarte-Gil C (2017). Diagnostic accuracy of nucleic acid amplification tests (NAATs) in urine for genitourinary tuberculosis: a systematic review and meta-analysis. BMC Infect Dis.

[48] Sun L, Chen Y, Yi P (2021). Serological detection of Mycobacterium tuberculosis complex infection in multiple hosts by One Universal ELISA. PLoS One.

[49] Yang H, Kruh-Garcia NA, Dobos KM (2012). Purified protein derivatives of tuberculin - past, present, and future. FEMS Immunol Med Microbiol.

[50] Ruiz-Tagle C, García P, Hernández M, Balcells ME (2024). Evaluation of concordance of new QuantiFERON-TB Gold Plus platforms for Mycobacterium tuberculosis infection diagnosis in a prospective cohort of household contacts. Microbiol Spectr.

[51] Kalokhe AS, Shafiq M, Lee JC (2013). Multidrug-resistant tuberculosis drug susceptibility and molecular diagnostic testing. Am J Med Sci.

[52] Naidoo K, Perumal R, Ngema SL, Shunmugam L, Somboro AM (2023). Rapid diagnosis of drug-resistant tuberculosis-opportunities and challenges. Pathogens.

[53] Hettick JM, Kashon ML, Simpson JP, Siegel PD, Mazurek GH, Weissman DN (2004). Proteomic profiling of intact mycobacteria by matrix-assisted laser desorption/ionization time-of-flight mass spectrometry. Anal Chem.

[54] Lin L, Chu H (2018). Quantifying publication bias in meta-analysis. Biometrics.

